# Identifying Novel Subtypes of Functional Gastrointestinal Disorder by Analyzing Nonlinear Structure in Integrative Biopsychosocial Questionnaire Data

**DOI:** 10.3390/jcm13102821

**Published:** 2024-05-10

**Authors:** Sa-Yoon Park, Hyojin Bae, Ha-Yeong Jeong, Ju Yup Lee, Young-Kyu Kwon, Chang-Eop Kim

**Affiliations:** 1 Department of Physiology, College of Korean Medicine, Gachon University, Seongnam 13120, Republic of Korea; sayou92@gachon.ac.kr (S.-Y.P.); jhyg1003@gmail.com (H.-Y.J.); 2 Biomedical Research Institute, Seoul National University Hospital, Seoul 03080, Republic of Korea; 3 Department of Physiology, College of Medicine, Seoul National University, Seoul 03080, Republic of Korea; dywl1210@snu.ac.kr; 4 Department of Internal Medicine, Keimyung University School of Medicine, Daegu 42601, Republic of Korea; leejygi@naver.com; 5 Division of Longevity and Biofunctional Medicine, School of Korean Medicine, Pusan National University, Yangsan 50612, Republic of Korea

**Keywords:** functional gastrointestinal disorders, UMAP, nonlinear clustering, subtype identification

## Abstract

**Background/Objectives:** Given the limited success in treating functional gastrointestinal disorders (FGIDs) through conventional methods, there is a pressing need for tailored treatments that account for the heterogeneity and biopsychosocial factors associated with FGIDs. Here, we considered the potential of novel subtypes of FGIDs based on biopsychosocial information. **Methods**: We collected data from 198 FGID patients utilizing an integrative approach that included the traditional Korean medicine diagnosis questionnaire for digestive symptoms (KM), as well as the 36-item Short Form Health Survey (SF-36), alongside the conventional Rome-criteria-based Korean Bowel Disease Questionnaire (K-BDQ). Multivariate analyses were conducted to assess whether KM or SF-36 provided additional information beyond the K-BDQ and its statistical relevance to symptom severity. Questions related to symptom severity were selected using an extremely randomized trees (ERT) regressor to develop an integrative questionnaire. For the identification of novel subtypes, Uniform Manifold Approximation and Projection and spectral clustering were used for nonlinear dimensionality reduction and clustering, respectively. The validity of the clusters was assessed using certain metrics, such as trustworthiness, silhouette coefficient, and accordance rate. An ERT classifier was employed to further validate the clustered result. **Results**: The multivariate analyses revealed that SF-36 and KM supplemented the psychosocial aspects lacking in K-BDQ. Through the application of nonlinear clustering using the integrative questionnaire data, four subtypes of FGID were identified: mild, severe, mind-symptom predominance, and body-symptom predominance. **Conclusions**: The identification of these subtypes offers a framework for personalized treatment strategies, thus potentially enhancing therapeutic outcomes by tailoring interventions to the unique biopsychosocial profiles of FGID patients.

## 1. Introduction

Functional gastrointestinal disorders (FGIDs) are chronic, heterogeneous intestinal tract illnesses characterized by a combination of gastrointestinal (GI) symptoms that cannot be explained by structural abnormalities, with functional dyspepsia (FD) and irritable bowel syndrome (IBS) being the most prevalent [1]. Despite not being life-threatening, FGIDs exhibit a negative impact on quality of life and work output and impose a high medical cost on both the individual and society, with more than 40% of people worldwide having FGIDs [2,3]. 

However, conventional medical treatment for FGIDs is generally ineffective [4], possibly due to a one-size-fits-all approach. Just a subset of patients whose symptoms are targeted by the relevant medications benefit from treatment [5], with only a 50–60% efficacy improvement [6]. Due to treatment difficulties and insufficient patient response to standard routine management of FGIDs, a novel and more effective management approach that considers FGIDs’ heterogeneity and biopsychosocial characteristics and provides tailored care is needed. 

Considering the heterogeneity of FGIDs, various approaches have been conducted to identify distinct subtypes. First, according to the Rome IV diagnostic criteria, the standard criteria for FGID [7], FGIDs are classified into 33 categories by anatomical location, including IBS and FD, with validated symptom-based criteria for each category [1]. These diagnostic criteria are primarily based on the most predominant symptoms. However, FGID patients who have different diagnoses frequently exhibit considerable overlap among symptoms [8,9,10]. Diverse multivariate approaches [11,12,13,14], including latent class analysis [15], have been utilized to better classify patients and improve treatment for these conditions. Despite their objective, data-driven methods, these approaches have only addressed the biological symptoms. To overcome these limitations of previous research, it is necessary to include not only biological symptoms, but also psychosocial aspects closely related to FGID among patients.

As reports that the complex interactions of environmental, psychological, and biological factors contribute to the development and maintenance of FGIDs increase, more attention is being paid to the biopsychosocial aspects of FGID. Thus, a more holistic-approach-based tailored treatment is required. One promising complementary strategy is traditional medicine. Traditional Korean medicine has almost three millennia of practice and treatment observation. Traditional Korean medicine emphasizes mind–body interaction and psychological pathogenicity, notably in stomach illnesses [16]. Patients receive personalized treatment based on their symptom patterns, a generalization of various symptoms and signs occurring at a certain stage of a disease, and the causes, pathogenesis, location, and type of the disease are investigated. Given the outstanding performance of traditional medicine, traditional medicine has already been used for diagnostic instruments and treatments [17,18]. 

Therefore, this study used the Rome-criteria-based Korean Bowel Disease Questionnaire (K-BDQ), a health-related quality of life questionnaire, the 36-item Short Form Health Survey (SF-36), and a traditional Korean medicine diagnosis questionnaire for digestive symptoms (KM). The Rome-criteria-based K-BDQ, while being a gold standard, might not encapsulate all of the multifaceted aspects of FGID. To complement the lacking psychosocial aspects, the KM and SF-36 were utilized. KM captured psychological and personality dimensions, as well as perspectives highly valued in traditional Korean medicine, which emphasizes a holistic approach (gathering information not just on digestive symptoms but on the entire body). The SF-36 is universally accepted for measuring health-related quality of life and encompasses psychological and social questions. By integrating these multifaceted biopsychosocial features of FGID, our objective was to delineate novel FGID subtypes through a data-driven exploration of the complex, nonlinear structure of FGID, using the graph-based dimensionality reduction method, UMAP, and the density-based clustering algorithm, spectral clustering.

## 2. Materials and Methods

### 2.1. Subjects

The 198 study subjects were enrolled from patients who visited the gastroenterology clinic at Keimyung University Dongsan Hospital in Daegu, Korea between March 2020 and February 2021. The inclusion criteria were as follows: provision of consent to participate in the study, aged 18–80 years, no organic problem in upper endoscopy and colonoscopy, and abdominal imaging studies (abdominal ultrasound or abdominopelvic computed tomography). The exclusion criteria were as follows: age < 18 or > 80 years, organic GI disease, such as malignancy, etc., previous gastrectomy, relevant comorbidities (e.g., moderate to severe cardiovascular, respiratory, hepatobiliary, pancreatic, renal, or neurologic disorder), recent use of medications that may affect GI symptoms (e.g., proton pump inhibitors, anti-histamine agents, or antibiotics), and pregnant or breastfeeding status among women. The study protocol was approved by the Institutional Review Board of Keimyung University Dongsan Medical Center (DSMC 2020-02-036). Informed consent was obtained from all study participants for the use of their medical records data.

### 2.2. Diagnostic Questionnaires

All subjects were requested to complete three types of questionnaires: the Rome-criteria-based K-BDQ, KM, and SF-36. Because the Rome-criteria-based questionnaire, the golden standard for diagnosis of FGID, was insufficient to provide psychosocial aspects of patients with FGID, KM and SF-36 were additionally collected. 

The Rome-criteria-based K-BDQ, a validated questionnaire translated from the original Bowel Disease Questionnaire, contains questions on upper and lower GI-related symptoms, mental symptoms, sociodemographic status, medical history, smoking, alcohol habits, marital status, educational level, and employment status [19,20,21]. For mental symptoms, the hospital anxiety depression scale score was used [22]. Among them, 14 questions regarding the presence of upper GI symptoms (e.g., dyspepsia, postprandial distress, epigastric pain, and reflux symptom), lower GI symptoms (e.g., abdominal pain associated with bowel movement, decreased bowel movement, hard or lumpy stool, a feeling of incomplete bowel movement, use of finger enema, and loose or watery stool) during the previous 6 months were analyzed [2]. Symptom severity was calculated by summing the severity scores of a total of 14 upper and lower GI symptom questions.

The KM questionnaire was made by combining four previously developed questionnaires: the spleen-qi-deficiency pattern questionnaire (SQDQ [23]), the scale for stomach-qi-deficiency pattern (SSQD [24]), the food retention questionnaire (FRQ [25]), and the personality questionnaire of the Sasang constitutional analysis tool (SCAT [26]). These questionnaires have been evaluated for their reliability and validity, and they have been employed in various research on GI diseases [27,28]. We constructed the KM questionnaire by combining these questionnaires and removing some duplicated questions (Supplementary Table S1).

SF-36 is a patient-reported questionnaire that measures health-related quality of life, and the version presented here is the RAND SF-36 [29]. The SF-36 covers eight health domains: physical functioning (10 items), bodily pain (2 items), role limitations due to physical health problems (4 items), role limitations due to personal or emotional problems (4 items), emotional well-being (5 items), social functioning (2 items), energy/fatigue (4 items), and general health perceptions (5 items). 

### 2.3. Data Preprocessing

All of the categorical variables were converted to numerical values via one-hot encoding. The get_dummies function of the Pandas package was used for the encoding process. All of the ordinal variables (Likert scale) were set such that the higher score represented the more severe condition. All of the variables were normalized between zero and one using the min–max scaler function of the Scikit-learn package [30]. As a result, a total of 167 variables comprised of 63 Rome-criteria-based K-BDQ variables, 65 KM variables, and 36 SF-36 variables (plus sex, age, and BMI) were used in this study. 

### 2.4. Comparison between Questionnaires 

#### 2.4.1. Canonical Correlation Analysis

In order to figure out whether KM and SF-36 obtain additional information over K-BDQ, we performed Canonical Correlation Analysis (CCA) on the Rome-criteria-based K-BDQ, KM, and SF-36. CCA is a multivariate statistical method that seeks a linear correlation between two sets of variables [31]. It enables a direct comparison of data groups, making it apt for our dataset consisting of dozens of questions.

#### 2.4.2. Multiple Linear Regression (MLR) 

To measure the quantity of additional information that KM and SF-36 have over K-BDQ at individual variable levels, MLR models were created for each questionnaire. In each MLR model, linear combinations of K-BDQ questions were used as explanatory variables, and each variable of SF-36 and KM was used as an outcome variable. Adjusted $R^{2}$ was used as the performance evaluation index of each model. 

### 2.5. Questionnaire Data-Based Clustering 

The graph-based dimensionality reduction methods, Uniform Manifold Approximation and Projection (UMAP [32,33]), and the density-based clustering algorithm, spectral clustering, were used for subtype classification. UMAP reduces dimension while maintaining the topological structure of the original dataset, i.e., retaining most of the data structure even in a reduced dimensional space, thus allowing for visualization and easier clustering of data. The spectral clustering algorithm clusters data points based on their similarity in the reduced space, making it efficient in identifying non-convex clusters. Their combined usage is crucial for uncovering inherent FGID subtypes. For hyperparameter optimization, we conducted a grid search for “n_neighbors” of UMAP (value of 3, 5, 10, 30, 50, and 100) and “n_clusters” of spectral clustering (value of 2, 3, 4, 5, and 6). The result is the average of 100 repeated measurements for each condition.

To evaluate the goodness of clustering analysis comprehensively, three types of metrics were used: trustworthiness, silhouette coefficient, and accordance rate. The n_neighbors and n_clusters were selected considering these three metrics altogether. The n_neighbors with the highest silhouette coefficient among those with a trustworthiness greater than 0.8 was selected. The n_clusters was selected among those with the optimized n_neighbors showing the highest accordance rate. However, to avoid the risk of overfitting, if the accordance rate was smaller than 0.8, the n_neighbors was changed to one that had a lower silhouette coefficient until the accordance rate was greater than 0.8 (Supplementary Table S2). 

Trustworthiness measures how well a datum’s structure is preserved after dimensionality reduction in a value between 0 and 1 [34].

$$Trustworthiness=1-\frac{2}{nk(2n-3k-1)}\sum_{i=1}^{n}\sum_{j\in U_{i}^{\left(k\right)}}\left(r\left(i,j\right)-k\right),$$

where $r\left(i,j\right)$ represents the rank of the data point j when the data vectors are ordered based on their Euclidean distance from the data vector i in the original data space, and $U_{i}^{\left(k\right)}$ represents the set of points that are among the k nearest neighbors in the low-dimensional space but not in the high-dimensional space. 

The silhouette coefficient measures the average value of how similar an object is to its own cluster (cohesion) compared to other clusters (separation) [35]. For one data point $i\in C_{I}$ (data point i in the cluster $C_{I}$),

$$Silhouette\;coefficient(i)=\;\frac{b(i)-a(i)}{max\{a\left(i\right),b\left(i\right)\}},$$

where $a\left(i\right)=\frac{1}{\left|C_{I}\right|-1}\sum_{j\in C_{I},i\neq j}d(i,j)$ and $b\left(i\right)=\underset{J\neq I}{\min}\frac{1}{\left|C_{J}\right|}\sum_{j\in C_{J}}d(i,j)$.

$a\left(i\right)$ is the mean distance between i and all other data points in the same cluster, i.e., intra-cluster distance, where $\left|C_{I}\right|$ is the number of points belonging to cluster $C_{I}$, and d(i,j) is the distance between data points i and j in the cluster $C_{I}$. $b\left(i\right)$ is the smallest mean distance of i to all points in any other cluster, i.e., the nearest-cluster distance. The silhouette coefficient is the mean Silhouette coefficient(i) across all data of the entire dataset. 

The accordance rate is a metric to evaluate generalizability, considering sample variability. The accordance rate is calculated using the adjusted mutual information score [36] between the assigned labels when using all of the samples (*n* = 198) and when using randomly selected smaller samples (*n* = 188). This metric was used to avoid overfitting the data samples and to provide generalizability of the clustering result. 

### 2.6. FGID-Related Feature Selection

To select clinically relevant variables indicating the severity of FGID, an extremely randomized tree (ERT) regressor algorithm was trained to predict the FGID symptom’s severity score. ERT regressor can provide the importance value of each variable for the trained model. For hyperparameter optimization, a randomized search on hyperparameters with nested cross-validation (double cross-validation [37]) was conducted to avoid data leakage. The hyperparameter configuration could be varied across the folds because the hyperparameters were tuned for each fold. Supplementary Table S3 summarizes the searched hyperparameters and their range for the ERT regressor model. 

The ERT regressor was trained and evaluated in a stratified k-fold cross-validation (k = 3) setting. The performance was evaluated using the coefficient of determination of the prediction $\left(R^{2}\right)$. 

### 2.7. Validation of the Identified Subtypes

To confirm the credibility of the clustered result, an ERT classifier was applied [38]. The ERT classifier was trained to decode each derived cluster label using the feature-selected questionnaire data. Hyperparameter optimization was conducted as in Supplementary Table S3.

The ERT classifier was trained and evaluated in stratified k-fold cross-validation (k = 2). The area under the receiver operating characteristic (AUROC) curve was calculated to evaluate how well the ML model distinguishes the subtypes with the questionnaire information of each patient. 

All of the analyses were conducted using the Scikit-learn Python package, except for the UMAP clustering and the trustworthiness calculation [33]. Throughout our analysis, appropriate statistical tests were employed to ascertain the significance of findings. For comparisons between two groups, the *t*-test was used. ANOVA with post hoc Tukey’s test was employed for comparing multiple groups. A *p*-value less than 0.05 was considered statistically significant.

## 3. Results

**Figure 1:**
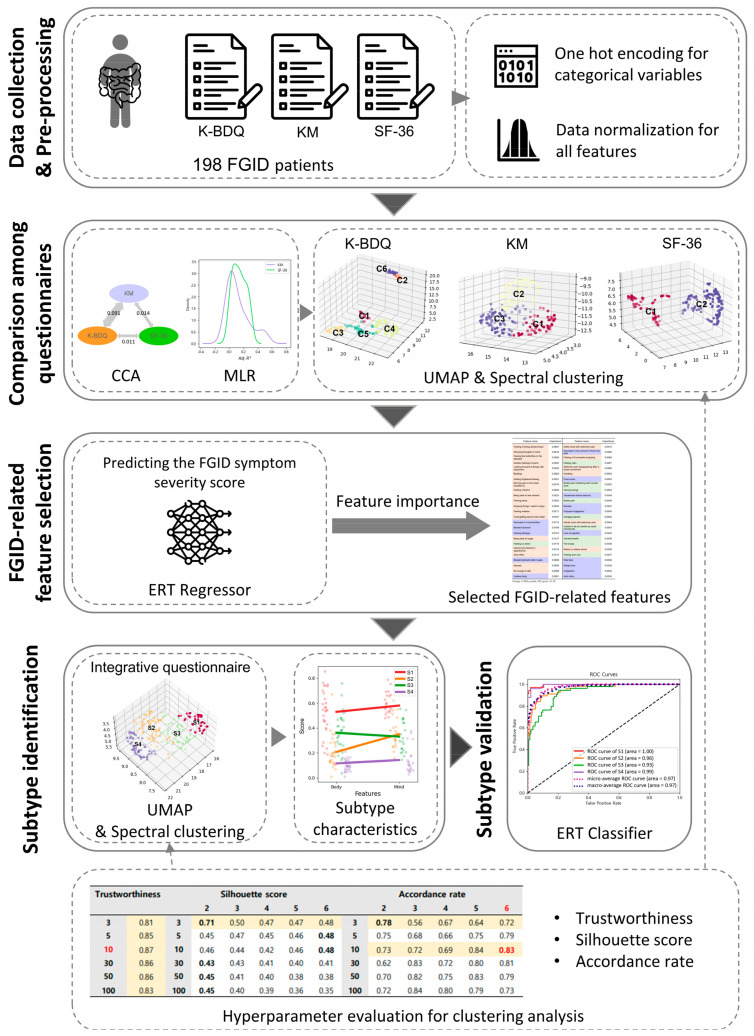
Overview of the whole analysis procedure. K-BDQ, Rome-criteria-based Korean Bowel Disease Questionnaire; KM, traditional Korean medicine diagnosis questionnaire for digestive symptoms; SF-36, 36-item Short Form Health Survey; CCA, Canonical Correlation Analysis; MLR, Multiple Linear Regression; FGID, functional gastrointestinal disorder; ERT, extremely randomized tree.

In the pursuit of a deeper understanding of FGID, this study aimed to delineate novel subtypes using an integrative questionnaire-based approach (Figure 1). We firstly delved into the comparative nuances of the three different questionnaires. Our initial analysis evaluated the synergistic information these questionnaires provide, with particular emphasis on discerning the supplementary value KM and SF-36 present relative to K-BDQ. Furthermore, to uncover the potential of KM and SF-36 questions in revealing crucial information for categorizing FGID subtypes, we conducted a nonlinear dimensionality reduction and clustering analysis using information from each questionnaire. Intriguingly, distinct clustered structures and their relevance to symptom severity emerged for each questionnaire, suggesting all three questionnaires’ effectiveness in identifying new FGID subtypes. To narrow down meaningful clinical information that contributes to the prediction of symptom severity, we trained an ERT regressor and selected the top 50 variables, thus making an integrative questionnaire. Leveraging the top 50 variables, we conducted dimensionality reduction and clustering analyses to identify novel FGID subtypes. Our findings underscored the existence of four distinct FGID subtypes, each characterized by a unique interplay of body and mind symptoms, including mild, severe, mind-symptom predominance, and body-symptom predominance subtypes. 

### 3.1. Assessing Complementary Information Obtained through KM and SF-36 Questionnaires Relative to K-BDQ

**Figure 2:**
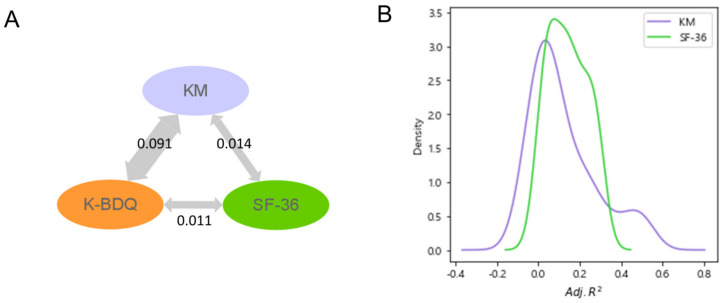
Exploring the similarity between each questionnaire. (**A**) Result of CCA between each pair of questionnaires, K-BDQ, KM, and SF-36. The width of the arrows represents the strength of the canonical correlation. (**B**) Distribution of adjusted $R^{2}$ values from MLR models to explain KM and SF-36 variables with a combination of upper and lower gastrointestinal symptom variables in K-BDQ. CCA, Canonical Correlation Analysis; MLR, Multiple Linear Regression; K-BDQ, Rome-criteria-based Korean Bowel Disease Questionnaire; KM, traditional Korean medicine diagnosis questionnaire for digestive symptoms; SF-36, 36-item Short Form Health Survey.

We quantified the correlations between KM, SF-36, and K-BDQ to determine if they provide more information than K-BDQ. K-BDQ and KM pairs, KM and SF-36 pairs, and K-BDQ and SF-36 pairs were initially compared using CCA. The K-BDQ and KM pair had the highest CCA score (0.0912), followed by the KM and SF-36 pair (0.0141) and the K-BDQ and SF-36 pair (0.0111). This result indicates that KM contains information obtained from both K-BDQ and SF-36 (Figure 2A). KM handles not only patients’ digestive symptoms but also aspects of their quality of life. 

To determine how much additional information KM and SF-36 provide over K-BDQ at individual variable levels, we built MLR models that describe each variable in KM and SF-36 with a combination of variables in K-BDQ and sorted the $R^{2}$ values in ascending order (Supplementary Table S4). Psychosocial and pattern identification questions were less explained by K-BDQ. The former includes questions like “Are your actions quick or slow?”, “Are you an extrovert or introvert?”, and “Are you active or passive?”, with adjusted $R^{2}$ values of −0.053, −0.032, and −0.025, respectively, while the latter includes “How many times do you urinate in the middle of the night?” and “Which do you dislike more, cold or heat?”, with adjusted $R^{2}$ values of −0.074 and −0.069, respectively. Physical functioning and general health SF-36 variables were less explained by K-BDQ factors. “Does your health now limit you in moderate activities, such as moving a table, pushing a vacuum cleaner, bowling, or playing golf?”, “How true or false is the following statement for you: ‘I expect my health to get worse’?”, and “Does your health now limit you in climbing several flights of stairs?” had adjusted $R^{2}$ values of 0.013, 0.024, and 0.031, respectively.

We observed the distribution of the adjusted $R^{2}$ values when each variable in KM and SF-36 was explained with a combination of K-BDQ variables using MLR models (Figure 2B). The difference between the two distributions was due to the heterogeneous question composition of the two questionnaires, KM and SF-36. The KM distribution’s broad range and bimodal form indicate the traditional-Korean-medicine-specific and various digestive-symptom-related questions. However, the comparatively narrow SF-36 distribution in the middle reflects the general questions focused on quality of life rather than digestive problems. These findings suggest that SF-36 and KM could supplement the psychosocial aspects lacking in the K-BDQ. 

### 3.2. Revealing the Relationship between Complementary Information from KM and SF-36 and FGID Symptom Severity through Nonlinear Clustering Analysis

**Figure 3:**
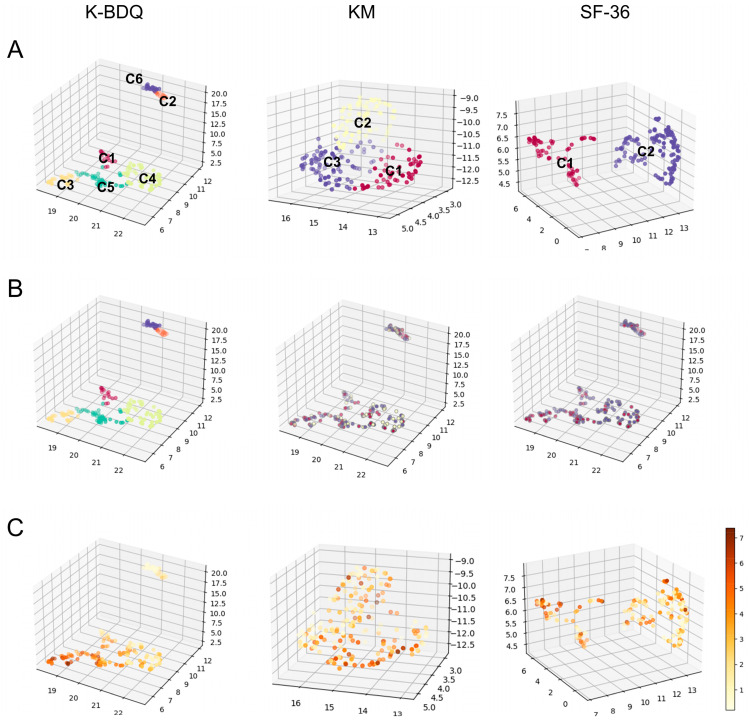
Relevance of the nonlinear clustered structure and the symptom severity. Each dot corresponds to a patient. Each axis corresponds to the UMAP dimensions 1, 2, and 3. (**A**) Result of dimensionality reduction and clustering analysis after hyperparameter tuning using each questionnaire’s information. Each color represents the result of clustering analysis. Patients’ data from K-BDQ, KM, and SF-36 were divided into six (n_neighbors = 10, n_clusters = 6, trustworthiness = 0.87, silhouette coefficient = 0.48, accordance rate = 0.83), three (n_neighbors = 30, n_clusters = 3, trustworthiness = 0.83, silhouette coefficient = 0.43, accordance rate = 0.84), and two (n_neighbors = 10, n_clusters = 2, trustworthiness = 0.87, silhouette coefficient = 0.64, accordance rate = 0.88) clusters. (**B**) Data embedding of K-BDQ was color-coded with the clustering result of 2A (the clustered labels when using K-BDQ, KM, and SF-36 information). Each color indicates the label colors of the clustering analysis (the same color as 2A). (**C**) Relevance of the clustered structure and the symptom severity when using K-BDQ, KM, and SF-36 information, respectively. Color intensity indicates the symptom severity. K-BDQ, Rome-criteria-based Korean Bowel Disease Questionnaire; KM, traditional Korean medicine diagnosis questionnaire for digestive symptoms; SF-36, 36-item Short Form Health Survey; C1, cluster 1; C2, cluster 2; C3, cluster 3.

To explore the possibility of KM and SF-36 questions being considered candidates for novel and meaningful clinical information required for the determination of the subtypes of FGID, we conducted a nonlinear dimensionality reduction and clustering analysis using information from each questionnaire and analyzed the relevance of the clustered structure and the symptom severity. UMAP [39] and the spectral clustering algorithm were employed for nonlinear dimensionality reduction and nonlinear clustering analysis. K-BDQ, KM, and SF-36 patient data were clustered into six, three, and two clusters, respectively (Figure 3A). Color-coding KM and SF-36 findings showed no grouping, indicating that each questionnaire had its own grouped structure (Figure 3B). Despite being obtained from the same patient samples, separate clustered structures were discovered, confirming that each questionnaire focused on different patient-related information.

Different aspects alone cannot be considered meaningful. The questions should be useful as medical information. To ensure that the clustered structure that the other two questionnaires capture contains medically meaningful information, we investigated the relevance of the clustered structure and the FGID symptom severity (Figure 3C). Symptom severity was calculated by adding the severity scores (from 0 to 1) of upper and lower GI symptoms of the K-BDQ. Except for one pair, the K-BDQ clusters differed significantly in symptom severity (one-way ANOVA with post hoc Tukey’s HSD test (*p* < 0.05), F = 130.10, *p* = 1.08 × 10^−59^, *p* = 0.9 for C1 compared with C4 and *p* < 0.05 for the rest of the pairs). The relevance between KM subtype results and symptom severity was partially significant (one-way ANOVA with post hoc Tukey’s HSD test (*p* < 0.05), F = 4.68, *p* = 0.01, *p* = 0.03 for C1 compared with C2, *p* = 0.01 for C1 compared with C3, *p* = 0.9 for C2 compared with C3). SF-36 subtype and symptom severity were significantly associated (*t*-test, T = 4.93, *p* = 2.54 × 10^−6^). 

Combining these, the nonlinear clustered structures were diverse, but they were related to FGID symptom severity. Therefore, all three questionnaires can be used to identify a novel subtype of FGID. 

### 3.3. Development of a Novel Integrative Questionnaire Using the Clinically Relevant Information 

To find meaningful clinical information that contributes to the prediction of symptom severity, we trained a regression model (ERT regressor) using all 167 variables in the three questionnaires (cross-validated mean train performance = 0.985, cross-validated mean test performance = 0.836). The top 50 feature importance variables that were highly related to the symptom severity were extracted (Table 1). Variables with higher importance were extracted from the Rome-criteria-based K-BDQ, as expected, given that the target variable was derived from the K-BDQ. Several KM and SF-36 variables were ranked highly, such as “Decrease in concentration”, “Bloated stomach”, and “Feeling lethargic” in KM and “Feeling so down” and “Feeling calm” in SF-36.

### 3.4. Novel Subtype Identification Using the Integrative Questionnaire

**Figure 4:**
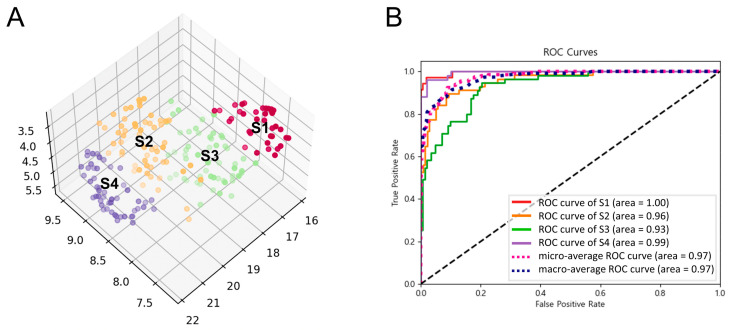
Subtype identification result of FGID patients using the integrative questionnaire information. (**A**) Result of dimensionality reduction and clustering analysis after hyperparameter tuning using the integrative questionnaire information. Patients’ data from the integrative questionnaire were divided into four (n_neighbors = 30, n_clusters = 4, trustworthiness = 0.84, silhouette coefficient = 0.39, accordance rate = 0.85) clusters. Each color indicates different subtypes. Each dot corresponds to a patient, and each axis corresponds to the UMAP dimensions 1, 2, and 3. (**B**) Receiver operating characteristic curves and corresponding area under the curve statistics for the prediction of subtype label based on the integrative questionnaire information. S1, subtype 1; S2, subtype 2; S3, subtype 3; S4, subtype 4.

To identify novel subtypes of FGID, we conducted the dimensionality reduction and cluster analysis to capture a data-driven, nonlinear structure behind our data using the top 50 variables (questions) that contributed to predicting the symptom severity. Four subtypes were derived as a result (Figure 4A). In addition, we confirmed the subtype identification result using a machine learning model (ERT classifier) for the subtype label classification. Two-fold cross-validation performance showed 0.97 for macro- and micro-average receiver operating characteristic curves (Figure 4B). 

**Figure 5:**
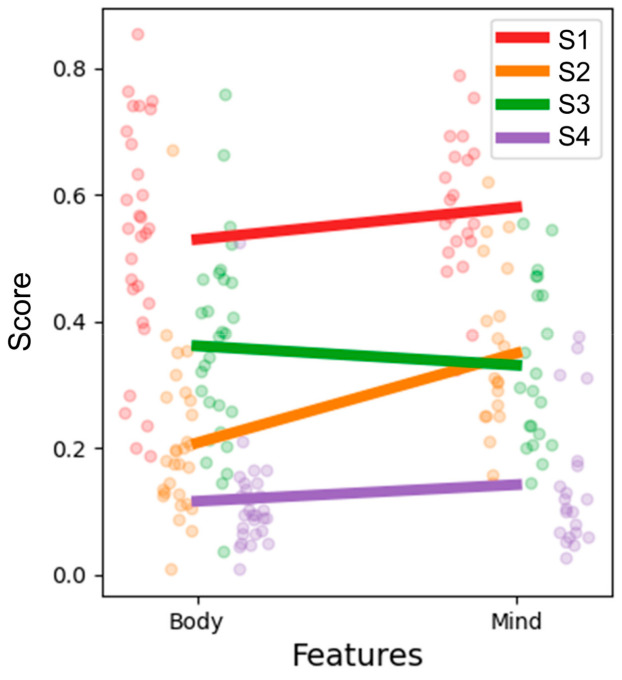
Characteristics of each FGID subtype. Line plot indicates the normalized average score of 29 body-symptom-related questions and 21 mind-symptom-related questions. Dot plot represents the normalized average score of each question for each subtype. S1, subtype 1; S2, subtype 2; S3, subtype 3; S4, subtype 4.

We explored the characteristics of each subtype. The top 50 variables were composed of 29 body-symptom-related questions and 21 mind-symptom-related questions. By calculating the normalized average score of these body and mind questions for each subtype, subtype 1 showed relatively higher scores for both body and mind questions, while subtype 4 showed relatively lower scores for both body and mind, contrastingly (Figure 5). Subtype 2 showed a higher mind score than the body score, while subtype 3 had a higher body score than mind score, suggesting the “mind-symptom predominance subtype” and the “body-symptom predominance subtype”, respectively. 

Some mind-symptom-related questions, such as “Worrying thoughts in mind”, “Feeling cheerful”, “Enjoying things I used to enjoy”, and “Having lost interest in appearance”, were among those for which subtype 2 (mind-symptom predominance subtype) had a higher score than subtype 3 (body-symptom predominance subtype) (Table 2). Some body-symptom-related questions, such as “Indigestion”, “Bloated stomach after meals”, “Decrease in the amount of food one eats”, and “General health”, were among the variables for which subtype 3 (body-symptom predominance subtype) had a normalized score of > 0.5. Subtype 1 showed the highest average score for all 50 questions, while subtype 4 showed the lowest average score for all 50 questions. Together, we identified novel subtypes of FGID using integrative questionnaire data.

Summing up, this study aimed to provide novel subtypes using an integrative questionnaire-based approach. Firstly, this study discerned the supplementary value KM and SF-36 present relative to K-BDQ. In addition, distinct clustered structures and their relevance to the symptom severity emerged for each questionnaire, suggesting that their collective use could improve FGID subtype identification by including comprehensive biopsychosocial information. This study proposed an integrative questionnaire providing meaningful clinical information that contributes to the prediction of symptom severity. Lastly, this approach uncovered four new FGID subtypes characterized by varying degrees of symptom severity and a complex interaction of physical and mental health components (mild, severe, mind-symptom predominance, and body-symptom predominance subtypes), paving the way for more personalized treatment strategies.

## 4. Discussion

In this study, we aimed to address the insufficient patient response to conventional management of FGID by identifying biopsychosocial-information-based novel subtypes using integrative questionnaire data. We observed that KM and SF-36 could supplement psychosocial aspects lacking in the Rome criteria. We found clinically relevant information contributing to the prediction of FGID severity among questions in these three questionnaires. The FGID-relevant clinical information contains psychosocial traits, such as “Decrease in concentration”, “Feeling lethargic”, “Feeling so down”, and “Feeling calm”. Nonlinear clustering analysis revealed four distinct FGID subtypes: mild, severe, mind-symptom predominance, and body-symptom predominance. This data-driven approach offers a new perspective on understanding and managing the complexity of FGID patients.

Compared to the current Rome IV classification, which is primarily based on symptoms in anatomic regions [7], our results suggested subtypes by considering more closely psychological factors, and they go beyond the limitation of symptom localization. The subtypes suggested in our study considered the severity between mind and body symptoms, rather than just the predominant symptoms. The importance of considering biopsychosocial factors in understanding and managing FGIDs has been increasingly recognized [40,41]. Recent studies have shown that patients with overlapping FGIDs exhibit higher symptom severity and psychosocial burden compared to those with single FGIDs [42], suggesting that the complex interplay of biological, psychological, and social factors contributes to the heterogeneity and severity of FGIDs. Emotional factors have long been linked to FGID symptoms, and the importance of emotional causes has grown [7,43]. In one study, 87% of FD patients had a diagnosis of psychosis compared to 25% of general dyspepsia patients [44]. In addition, a structural equation modeling study of 259 FGID patients found statistical significance between depression, somatization, gastric emptying, and the existing empirically derived symptom-based subgroups [45]. In conclusion, our findings align with the growing evidence supporting the biopsychosocial model in FGIDs and offer a novel approach to subtype identification based on integrative patient data.

For reliable subtype identification, we utilized nonlinear dimensionality reduction and nonlinear clustering analysis. UMAP is a recently proposed nonlinear graph-based dimensionality reduction method, which is known for improving clustering accuracy by seeking to accurately represent local structure and to better incorporate global structure [39]. There have been many successful cases of UMAP applications in medical fields [46,47,48]. Meanwhile, there have been concerns regarding the reliability of the results due to the unsupervised nature of the procedure. To ensure the credibility of our findings, we employed a two-step validation approach. First, we introduced the accordance rate, which assesses the consistency of clustering results across multiple iterations and when applied to randomly selected subsets of the dataset. A high accordance rate indicates the reliability of the clustering results and mitigates potential variability arising from limited dataset sizes. Second, we utilized the ERT classifier, a supervised machine learning algorithm, to validate the distinctness of the identified clusters. A high AUROC score demonstrates that the identified subtypes are indeed distinct and not merely an artifact of the clustering algorithm. While the sample size of our study (*n* = 198) may be considered modest, the rigorous statistical methods employed effectively address concerns regarding the robustness and reliability of our results.

Our research is limited in that the investigation is based on patient-reported symptoms rather than objective physiological information, such as biomarkers. However, considering that the diagnosis of FGID is highly dependent on the Rome-criteria-based questionnaire worldwide [49], we believe that our research on questionnaire-data-based identification of subtypes can be of great help. Especially in some countries, such as the U.S., *Helicobacter pylori* tests and endoscopies can be performed only when symptom-based drug treatment fails [50]. In addition, the current study is limited by its small sample size of *n* = 198. Despite the limited circumstances of the small sample size, we tried to provide credibility by double-validating the identified subtype result by confirming the accordance rate and the classification performance of the ERT classifier. Nevertheless, future clinical research with an increased sample size is required to provide a more robust result.

As a possible future study, a follow-up study with the participating patients in this study would be worthwhile. Comparison of disease progress, gut microbiome, and metabolite distribution for each subtype would be useful for validating the reliability of the presented subtypes, which might allow for the discovery of biomarkers for subtype diagnosis or provide insights for tailored treatment. Furthermore, using the developed integrative questionnaire and trained subtype classification model, we can predict the subtypes of unseen FGID patients.

## 5. Conclusions

We discovered important clinical information regarding FGID using multidimensional patient-reported questionnaires, the Rome-criteria-based K-BDQ, SF-36, and KM, suggesting the biopsychosocial integrative questionnaire. We developed a reliable metric for evaluating nonlinear dimensionality reduction and clustering analyses. Based on our analysis using the metric, we observed four potential FGID subtypes—mild, severe, mind-symptom predominance, and body-symptom predominance. While further validations with larger sample sizes and external datasets are necessary to solidify these findings, these subtypes provide a promising avenue for personalized treatment considerations.

## Figures and Tables

**Table 1:** Top 50 features showing high importance in the regressor model with data from multiple questionnaires.

Feature Name	Importance	Feature Name	Importance
Feeling of being slowed down	0.0997	Softer stool with abdomen pain	0.0074
Worrying thoughts in mind	0.0918	Decrease in the amount of food one eats	0.0069
Feeling like butterflies in the stomach	0.0559	Feeling of incomplete emptying	0.0066
Sudden feelings of panic	0.0450	Feeling calm	0.0061
Looking forward to things with enjoyment	0.0425	Abdomen pain disappearing after a bowel movement	0.0060
Bloating	0.0363	Vomiting	0.0054
Getting frightened feeling	0.0351	Food coma	0.0053
Burning pain in the chest (heartburn)	0.0316	Bodily pain interfering with normal work	0.0053
Feeling cheerful	0.0285	Having energy	0.0052
Being able to feel relaxed	0.0255	Tenderness below sternum	0.0049
Feeling tense	0.0253	Bodily pain	0.0048
Enjoying things I used to enjoy	0.0250	Nausea	0.0047
Feeling restless	0.0217	Frequent indigestion	0.0045
Food getting stuck in the chest	0.0191	Unhappy person	0.0045
Decrease in concentration	0.0172	Harder stool with abdomen pain	0.0044
Bloated stomach	0.0169	Unable to be as careful as usual (emotional)	0.0043
Feeling lethargic	0.0157	Loss of appetite	0.0043
Being able to laugh	0.0127	General health	0.0039
Feeling so down	0.0119	Full of pep	0.0038
Having lost interest in appearance	0.0119	Mushy or watery stools	0.0038
Acid reflux	0.0115	Feeling worn out	0.0037
Bloated stomach after meals	0.0099	Pale face	0.0036
Nausea	0.0095	Weight loss	0.0035
No energy to talk	0.0088	Indigestion	0.0035
Listless body	0.0081	Acid reflux	0.0034

Orange, K-BDQ; purple, KM; green, SF-36.

**Table 2:** Normalized average score of each subtype for the mind and body variables (top 50 variables).

	Variable Name	S1	S2	S3	S4
**Body-symptom-related questions**	**Feeling of being slowed down**	0.60	0.13	**0.48**	0.10
	Bloating	0.63	0.20	0.39	0.10
	Burning pain in the chest (heartburn)	0.59	0.13	0.38	0.09
	Feeling of being slowed down	0.75	0.38	0.41	0.17
	Nausea	0.40	0.07	0.16	0.05
	Acid reflux	0.47	0.11	0.27	0.06
	Softer stool with abdomen pain	0.39	0.13	0.23	0.09
	Harder stool with abdomen pain	0.20	0.09	0.20	0.10
	Vomiting	0.26	0.01	0.04	0.01
	Mushy or watery stools	0.28	0.14	0.15	0.09
	Abdomen pain disappearing after a bowel movement	0.24	0.14	0.21	0.10
	Feeling of incomplete emptying	0.19	0.11	0.18	0.05
	**Indigestion**	0.76	0.20	**0.66**	0.21
	Bloated stomach	0.74	0.28	0.48	0.15
	Feeling lethargic	0.70	0.25	0.42	0.12
	**Bloated stomach after meals**	0.74	0.29	**0.52**	0.16
	Listless body	0.74	0.35	0.47	0.15
	Tenderness below sternum	0.55	0.18	0.47	0.13
	**Decrease in the amount of food one eats**	0.68	0.36	**0.55**	0.17
	No energy to talk	0.57	0.21	0.29	0.05
	Nausea	0.50	0.11	0.27	0.08
	Pale face	0.46	0.17	0.35	0.05
	Weight loss	0.43	0.32	0.38	0.07
	Acid reflux	0.55	0.21	0.33	0.14
	Frequent indigestion	0.54	0.20	0.41	0.13
	Food coma	0.53	0.18	0.32	0.10
	Bodily pain	0.57	0.28	0.46	0.12
	Bodily pain interfering with normal work	0.45	0.18	0.26	0.07
	**General health**	0.85	0.67	**0.76**	0.53
**Mind-symptom-related questions**	*Worrying thoughts in mind*	0.63	**0.36**	0.35	0.12
	*Feeling cheerful*	0.69	**0.54**	0.44	0.17
	*Enjoying things I used to enjoy*	0.60	**0.40**	0.24	0.05
	Feeling like butterflies in the stomach	0.53	0.25	0.27	0.05
	*Having lost interest in appearance*	0.48	**0.37**	0.21	0.07
	Feeling tense	0.56	0.27	0.30	0.10
	*Looking forward to things with enjoyment*	0.56	**0.30**	0.21	0.06
	*Sudden feelings of panic*	0.53	**0.31**	0.29	0.08
	*Getting frightened feeling*	0.51	**0.25**	0.24	0.07
	*Being able to feel relaxed*	0.59	**0.41**	0.38	0.18
	*Being able to laugh*	0.38	**0.25**	0.15	0.10
	Feeling restless	0.32	0.15	0.18	0.03
	Decrease in concentration	0.69	0.34	0.48	0.12
	Loss of appetite	0.56	0.31	0.47	0.11
	Unable to be as careful as usual (emotional)	0.67	0.16	0.20	0.06
	Feeling worn out	0.49	0.29	0.32	0.14
	**Having energy**	0.76	0.55	**0.56**	0.32
	Feeling so down	0.54	0.21	0.22	0.13
	***Full of pep***	0.79	**0.62**	**0.55**	0.38
	*Feeling calm*	0.66	**0.48**	0.47	0.31
	*Unhappy person*	0.66	**0.51**	0.44	0.36

Orange color: K-BDQ; purple color: KM; green color: SF-36. Bold face in feature name: normalized average score of S3 > 0.5; italic face in feature name: normalized average score of S2 > S3. S1, subtype 1; S2, subtype 2; S3, subtype 3; S4, subtype 4.

## Data Availability

The datasets generated and/or analyzed during this study are available from the corresponding author upon reasonable request.

## Supplementary Materials

The following supporting information can be downloaded at: https://www.mdpi.com/article/10.3390/jcm13102821/s1, Table S1: KM questionnaire; Table S2: Hyperparameter tuning using trustworthiness, silhouette coefficient, and accordance rate; Table S3: Grid search space for the hyperparameter optimization for the ERTR and ERT models; Table S4: $R^{2}$ values of KM and SF-36 variables arranged in an ascending order.

## References

[1] Black C.J., Drossman D.A., Talley N.J., Ruddy J., Ford A.C. (2020). Functional gastrointestinal disorders: Advances in understanding and management. Lancet.

[2] Sperber A.D., Bangdiwala S.I., Drossman D.A., Ghoshal U.C., Simren M., Tack J., Whitehead W.E., Dumitrascu D.L., Fang X., Fukudo S. (2021). Worldwide prevalence and burden of functional gastrointestinal disorders, results of Rome Foundation Global Study. Gastroenterology.

[3] Xie S., Zhang H., Liu Y., Gao K., Zhang J., Fan R., Xie S., Xie Z., Wang F., Jiang W. (2022). The Role of Serum Metabolomics in Distinguishing Chronic Rhinosinusitis with Nasal Polyp Phenotypes. Frontiers.

[4] Van Oudenhove L., Levy R.L., Crowell M.D., Drossman D.A., Halpert A.D., Keefer L., Lackner J.M., Murphy T.B., Naliboff B.D. (2016). Biopsychosocial Aspects of Functional Gastrointestinal Disorders. Gastroenterology.

[5] Chang L., Di Lorenzo C., Farrugia G., Hamilton F.A., Mawe G.M., Pasricha P.J., Wiley J.W. (2018). Functional bowel disorders: A roadmap to guide the next generation of research. Gastroenterology.

[6] Chey W.D. (2017). Symposium report: An evidence-based approach to Ibs and Cic: Applying new advances to daily practice: A review of an adjunct clinical symposium of the American College of Gastroenterology Meeting October 16, 2016• Las Vegas, Nevada. Gastroenterol. Hepatol..

[7] Drossman D.A. (2016). Functional gastrointestinal disorders: History, pathophysiology, clinical features, and Rome IV. Gastroenterology.

[8] Frissora C.L., Koch K.L. (2005). Symptom overlap and comorbidity of irritable bowel syndrome with other conditions. Curr. Gastroenterol. Rep..

[9] Talley N.J., Dennis E.H., Schettler-Duncan V.A., Lacy B.E., Olden K.W., Crowell M.D. (2003). Overlapping upper and lower gastrointestinal symptoms in irritable bowel syndrome patients with constipation or diarrhea. Am. J. Gastroenterol..

[10] Talley N.J. (2006). A unifying hypothesis for the functional gastrointestinal disorders: Really multiple diseases or one irritable gut?. Rev. Gastroenterol. Disord..

[11] Van Oudenhove L., Holvoet L., Vandenberghe J., Vos R., Tack J. (2011). Do we have an alternative for the Rome III gastroduodenal symptom-based subgroups in functional gastroduodenal disorders? A cluster analysis approach. Neurogastroenterol. Motil..

[12] Westbrook J., Talley N. (2002). Empiric clustering of dyspepsia into symptom subgroups: A population-based study. Scand. J. Gastroenterol..

[13] Talley N.J., Holtmann G., Agréus L., Jones M. (2000). Gastrointestinal symptoms and subjects cluster into distinct upper and lower groupings in the community: A four nations study. Am. J. Gastroenterol..

[14] Koloski N., Jones M., Young M., Talley N. (2015). Differentiation of functional constipation and constipation predominant irritable bowel syndrome based on Rome III criteria: A population-based study. Aliment. Pharmacol. Ther..

[15] Zinsmeister A.R., Herrick L.M., Loftus Y.A.S., Schleck C.D., Talley N.J. (2018). Identification and validation of functional gastrointestinal disorder subtypes using latent class analysis: A population-based study. Scand. J. Gastroenterol..

[16] Cao J., Ding L. (2022). Psychosomatic Practice in Gastroenterology: New Insights and Models from China. Psychother. Psychosom..

[17] Kim Y.S., Kim J.-W., Ha N.-Y., Kim J., Ryu H.S. (2020). Herbal therapies in functional gastrointestinal disorders: A narrative review and clinical implication. Front. Psychiatry.

[18] Sankararaman S., Velayuthan S., Chen Y., Robertson J., Sferra T.J. (2022). Role of traditional Chinese herbal medicines in functional gastrointestinal and motility disorders. Curr. Gastroenterol. Rep..

[19] Choi Y.J., Hwang S.W., Kim N., Park J.H., Oh J.C., Lee D.H. (2014). Association between SLC6A4 serotonin transporter gene lainked polymorphic region and ADRA2A− 1291C>G and irritable bowel syndrome in Korea. J. Neurogastroenterol. Motil..

[20] Hwang S.W., Kim N., Jung H.K., Park J.H., Choi Y.J., Kim H., Kim J., Kim J.S., Jung H.C. (2014). The association of SLC 6 A 4 5-HTTLPR and TRPV1 945 G>C with functional dyspepsia in K orea. J. Gastroenterol. Hepatol..

[21] Noh Y.W., Jung H.-K., Kim S.-E., Jung S.-A. (2010). Overlap of erosive and non-erosive reflux diseases with functional gastrointestinal disorders according to Rome III criteria. J. Neurogastroenterol. Motil..

[22] Oh S.-M., Min K.-J., Park D.-B. (1999). A study on the standardization of the hospital anxiety and depression scale for Koreans: A comparison of normal, depressed and anxious groups. J. Korean Neuropsychiatr. Assoc..

[23] Oh H.-W., Lee J.-W., Kim J.-S., Song E.-Y., Shin S.-W., Han G.-J., Lu H., Lee J.-H. (2014). Study on the development of a standard instrument of diagnosis and assessment for spleen Qi deficiency pattern. J. Korean Med..

[24] Lee J., Park J.-W., Ko S.-J., Kim J. (2018). Development and validation of a new pattern identification scale for Stomach Qi Deficiency. Eur. J. Integr. Med..

[25] Park Y.-J., Lim J.-S., Park Y.-B. (2013). Development of a valid and reliable food retention questionnaire. Eur. J. Integr. Med..

[26] Do J.-H., Jang E., Ku B., Jang J.-S., Kim H., Kim J.Y. (2012). Development of an integrated Sasang constitution diagnosis method using face, body shape, voice, and questionnaire information. BMC Complement. Altern. Med..

[27] Kim J., Kim J., Kim J., Kim K.H., Kim J., Kim J., Kim J., Kim K.H. (2015). Reliability and validity analysis of a standard instrument of diagnosis and assessment for spleen Qi deficiency pattern in chronic dyspepsia patients. J. Korean Med..

[28] Lee J., Yim M.H., Kim J.Y. (2018). Test-retest reliability of the questionnaire in the Sasang constitutional analysis tool (SCAT). Integr. Med. Res..

[29] Ware J.E., Sherbourne C.D. (1992). The MOS 36-item short-form health survey (SF-36). I. Conceptual framework and item selection. Med. Care.

[30] Pedregosa F., Varoquaux G., Gramfort A., Michel V., Thirion B., Grisel O., Blondel M., Prettenhofer P., Weiss R., Dubourg V. (2011). Scikit-learn: Machine learning in Python. J. Mach. Learn. Res..

[31] Hotelling H. (1992). Relations between two sets of variates. Breakthroughs in Statistics.

[32] Becht E., McInnes L., Healy J., Dutertre C.-A., Kwok I.W., Ng L.G., Ginhoux F., Newell E.W. (2019). Dimensionality reduction for visualizing single-cell data using UMAP. Nat. Biotechnol..

[33] McInnes L., Healy J., Melville J. (2018). Umap: Uniform manifold approximation and projection for dimension reduction. arXiv.

[34] Venna J., Kaski S. (2001). Neighborhood preservation in nonlinear projection methods: An experimental study. Proceedings of the International Conference on Artificial Neural Networks.

[35] Rousseeuw P.J. (1987). Silhouettes: A graphical aid to the interpretation and validation of cluster analysis. J. Comput. Appl. Math..

[36] Xuan N., Julien V., Wales S., Bailey J. (2010). Information theoretic measures for clusterings comparison: Variants, properties, normalization and correction for chance. Prop. Norm. Correct. Chance.

[37] Krstajic D., Buturovic L.J., Leahy D.E., Thomas S. (2014). Cross-validation pitfalls when selecting and assessing regression and classification models. J. Cheminformatics.

[38] Geurts P., Ernst D., Wehenkel L. (2006). Extremely randomized trees. Mach. Learn..

[39] Allaoui M., Kherfi M.L., Cheriet A. (2020). Considerably improving clustering algorithms using UMAP dimensionality reduction technique: A comparative study. Proceedings of the International Conference on Image and Signal Processing.

[40] Fikree A., Byrne P. (2021). Management of functional gastrointestinal disorders. Clin. Med..

[41] Aboubakr A., Cohen M.S. (2021). Functional bowel disease. Clin. Geriatr. Med..

[42] Berens S., Engel F., Gauss A., Tesarz J., Herzog W., Niesler B., Stroe-Kunold E., Schaefert R. (2020). Patients with multiple functional gastrointestinal disorders (FGIDs) show increased illness severity: A cross-sectional study in a tertiary care FGID specialty clinic. Gastroenterol. Res. Pract..

[43] Bennett E., Piesse C., Palmer K., Badcock C., Tennant C., Kellow J. (1998). Functional gastrointestinal disorders: Psychological, social, and somatic features. Gut.

[44] Varis K. (1987). Psychosomatic factors in gastrointestinal disorders. Ann. Clin. Res..

[45] Franks I. (2012). Psychological factors in functional dyspepsia—Keeping an open mind. Nat. Rev. Gastroenterol. Hepatol..

[46] Sánchez-Rico M., Alvarado J.M. (2019). A machine learning approach for studying the comorbidities of complex diagnoses. Behav. Sci..

[47] Yang Y., Sun H., Zhang Y., Zhang T., Gong J., Wei Y., Duan Y.-G., Shu M., Yang Y., Wu D. (2021). Dimensionality reduction by UMAP reinforces sample heterogeneity analysis in bulk transcriptomic data. Cell Rep..

[48] Weijler L., Kowarsch F., Wödlinger M., Reiter M., Maurer-Granofszky M., Schumich A., Dworzak M.N. (2022). UMAP Based Anomaly Detection for Minimal Residual Disease Quantification within Acute Myeloid Leukemia. Cancers.

[49] Miwa H., Kusano M., Arisawa T., Oshima T., Kato M., Joh T., Suzuki H., Tominaga K., Nakada K., Nagahara A. (2015). Evidence-based clinical practice guidelines for functional dyspepsia. J. Gastroenterol..

[50] Aziz I., Palsson O.S., Törnblom H., Sperber A.D., Whitehead W.E., Simrén M. (2018). Epidemiology, clinical characteristics, and associations for symptom-based Rome IV functional dyspepsia in adults in the USA, Canada, and the UK: A cross-sectional population-based study. Lancet Gastroenterol. Hepatol..

