# Supplementary material for: Identifying Novel Subtypes of Functional Gastrointestinal Disorder by Analyzing Nonlinear Structure in Integrative Biopsychosocial Questionnaire Data
Source: J Clin Med. 2024 May 10;13(10):2821. doi: 10.3390/jcm13102821 (PMC11122158; doi:10.3390/jcm13102821)
Supplement: Supplementary file 1 [file jcm-13-02821-s001.zip › jcm-2951000-supplementary.pdf]

Supplementary Table S1. KM questionnaire.

	Questionnaire
1	My stool is loose.
2	I feel bloated or full without eating much, and it gets worse after eating.
3	I feel listless and have no energy throughout my whole body.
4	The amount of food I eat has decreased.
5	My face has been pale or yellowish.
6	I don't have energy to talk, or I lose energy when I talk a lot.
7	I've lost weight.
8	I tend to easily get tired, or I lose concentration or motivation easily.
9	I have lost my appetite.
10	I have a discomfort in the upper stomach, but it gets better when it gets warm.
11	I often burp.
12	I don't know the taste of food.
13	I feel pain when I press below the sternum.
14	I feel bloated after meals.
15	I feel tired and don't want to move.
16	I urinate frequently.
17	My body swells easily.
18	I have pain in the joints.
19	I frequently suffer from indigestion.
20	I have a traveler's diarrhea.
21	I'm allergic to certain ingredients.
22	I have a stomachache right after eating.
23	I defecate right after eating.
24	I tend to deliver loose stools.
25	I gain weight gradually.
26	I fall into food coma.
27	I feel sick and nauseous.
28	I have an acid reflux
29	When did the symptoms appear?
30	Are you bold or delicate?
31	Are your actions quick or slow?
32	Are you active or passive?
33	Are you an extrovert or introvert?
34	Are you masculine or feminine?
35	Do you get excited or remain calm?
36	Do you digest well?
37	How is your appetite?

38	How much do you perspire?
39	How do you feel after perspiration?
40	Is it challenging to hold it in when the urge to defecate arises?
41	How many times do you urinate in the middle of the night?
42	Which do you dislike more, cold or heat?
43	How is the temperature of your drinking water?

Supplementary Table S2. Hyperparameter tuning using trustworthiness, silhouette coefficient, and accordance rate (average values of 100 times repeated).

ROME

Trustworthiness			Silhouette score						Accordance rate				
			2	3	4	5	6		2	3	4	5	6
3	0.81	3	**0.71**	0.50	0.47	0.47	0.48	3	**0.78**	0.56	0.67	0.64	0.72
5	0.85	5	0.45	0.47	0.45	0.46	**0.48**	5	0.75	0.68	0.66	0.75	0.79
10	0.87	10	0.46	0.44	0.42	0.46	**0.48**	10	0.73	0.72	0.69	0.84	0.83
30	0.86	30	**0.43**	0.43	0.41	0.40	0.41	30	0.62	0.83	0.72	0.80	0.81
50	0.86	50	**0.45**	0.41	0.40	0.38	0.38	50	0.70	0.82	0.75	0.83	0.79
100	0.83	100	**0.45**	0.40	0.39	0.36	0.35	100	0.72	0.84	0.80	0.79	0.73

KM

			2	3	4	5	6		2	3	4	5	6
3	0.82	3	0.44	0.42	0.47	**0.48**	0.47	3	0.30	0.47	0.61	**0.64**	0.63
5	0.86	5	0.36	**0.44**	0.40	0.39	0.41	5	0.58	0.67	0.66	0.67	0.67
10	0.87	10	0.37	**0.45**	0.39	0.38	0.39	10	0.54	0.76	0.65	0.76	0.70
30	0.83	30	0.34	**0.43**	0.35	0.34	0.36	30	0.53	0.84	0.74	0.69	0.75
50	0.83	50	0.33	**0.40**	0.35	0.34	0.34	50	0.69	0.82	0.72	0.71	0.74
100	0.77	100	0.31	0.38	0.32	0.31	0.31	100	0.60	0.78	0.58	0.59	0.54

SF-36

			2	3	4	5	6		2	3	4	5	6
3	0.80	3	0.45	0.52	**0.57**	0.55	0.56	3	0.26	0.53	0.72	0.66	0.71
5	0.86	5	0.58	**0.62**	0.54	0.55	0.56	5	0.74	0.79	0.74	0.83	0.84
10	0.87	10	**0.64**	0.54	0.52	0.52	0.51	10	0.88	0.77	0.83	0.85	0.86
30	0.87	30	**0.62**	0.51	0.45	0.42	0.44	30	0.82	0.87	0.75	0.75	0.88
50	0.86	50	**0.58**	0.48	0.40	0.38	0.41	50	0.84	0.84	0.76	0.81	0.86
100	0.81	100	**0.54**	0.45	0.38	0.36	0.39	100	0.90	0.85	0.82	0.83	0.86

Integrative questionnaire

			2	3	4	5	6		2	3	4	5	6
3	0.80	3	0.711	0.40	0.47	0.48	0.47	3	0.75	0.54	0.59	0.61	0.65
5	0.84	5	**0.491**	0.41	0.40	0.41	0.43	5	0.67	0.67	0.70	0.71	0.74
10	0.84	10	**0.485**	0.41	0.40	0.39	0.40	10	0.76	0.77	0.78	0.73	0.73
30	0.84	30	**0.494**	0.42	0.39	0.37	0.35	30	0.81	0.81	0.85	0.79	0.71
50	0.84	50	**0.491**	0.41	0.38	0.36	0.35	50	0.79	0.80	0.81	0.75	0.74
100	0.85	100	**0.483**	0.40	0.36	0.35	0.34	100	0.83	0.83	0.82	0.73	0.67

Supplementary Table S3. Grid search space for the hyperparameter optimization for the ERTR and ERT models.

Model	Hyperparameter	Haperparameter values
ERT	n_estimators	200, 400, 600, 800, 1000, 1200, 1400, 1600, 1800, 2000
	max_depth	10, 20, 30, 40, 50, 60, 70, 80, 90, 100, 110, None
	min_samples_leaf	1, 2, 4
	min_samples_split	2, 5, 10

ERTR, extremely randomized tree regressor; ERT, extremely randomized trees

Supplementary Table S4. R^2 values of KM and SF-36 variables arranged in an ascending order.

Feature name	R^2	Feature name	R^2
times to urinate at night_1	-0.07412	urinate frequently	0.06894
sensitive to the heat or cold_1	-0.06943	Don't sweat_2	0.074804
easygoing_1	-0.05301	feeling after sweating_1	0.079494
times to urinate at night_2	-0.04715	water temperature_0	0.08653
sensitive to the heat or cold_2	-0.04715	difficult to hold it in_0	0.094738
sensitive to the heat or cold_0	-0.0366	difficult to hold it in_2	0.094738
easygoing_0	-0.0319	body swell up	0.098256
introvert_0	-0.0319	water temperature_2	0.100601
easygoing_2	-0.02721	rational_0	0.106464
timid_0	-0.02487	stomachache after meals	0.127571
sensitive_0	-0.01666	can't taste food	0.134607
timid_1	-0.0108	no appetite_2	0.136952
indigestion_1	-0.00845	indigestion_0	0.155714
times to urinate at night_0	-0.00493	indigestion_2	0.176821
dont sweat_0	-0.00376	bowel movements right after meals	0.177994
introvert_1	-0.00142	belched frequently	0.181512
rational_2	-0.00142	lose weight	0.212
food hives	0.006792	indigestion frequently	0.213173
rational_1	0.006792	pain relieved by warming up	0.229589
womanly_0	0.01031	portions get smaller	0.23897
water temperature_1	0.013827	lose appetite	0.240143
womanly_1	0.018518	traveler's diarrhea	0.241315
sensitive_1	0.026726	tenderness below sternum	0.260077
sensitive_2	0.026726	food coma	0.272976
no appetite_1	0.029071	look pale	0.303464
womanly_2	0.044315	regurgitate acid	0.30581

timid_2	0.045488	soft stool	0.358577
feeling after sweating_0	0.046661	no energy to talk	0.396101
no appetite_0	0.047833	feel lethargic	0.400792
feeling after sweating_2	0.049006	low on energy	0.451214
introvert_2	0.05956	nausea.1	0.46294
difficult to hold it in_1	0.05956	feel bloated	0.50281
dont sweat_1	0.063077	mushy stool	0.503982
joint aching	0.065423	demotivated	0.503982
gain weight	0.065423	feel bloated after meals	0.509845
onset of symptoms	0.065423		
physical health_accomplished less	-0.00728	how much time interfere with social activities	0.129917
moderate activities	0.012655	walking one block	0.141643
physical health_cut down time	0.018518	get sick easier	0.153369
expect health worse	0.024381	health excellent	0.163923
climbing several flights of stairs	0.031417	general health_compared to 1 year ago	0.180339
physical health_took extra effort	0.043143	normal social activities	0.182685
vigorous activities	0.045488	feel calm	0.18972
physical health_limited in the work	0.051351	feel worn out	0.199101
walking more than a mile	0.056042	feel so down	0.215518
lifting or carrying groceries	0.060732	bodily pain	0.226071
bathing or dressing yourself	0.06425	general health	0.23897
emotional_cut down time	0.066595	happy person	0.253042
emotional_accomplished less	0.083012	nervous person	0.263595
walking several blocks	0.1135	pain interfere with normal work	0.264768
halthy as anybody	0.117018	feel downhearted	0.282357

Purple, KM; green, SF-36. R^2; adjusted R^2 value.
